# The Influence of Plasma Prekallikrein Oligonucleotide Antisense Therapy on Coagulation and Fibrinolysis Assays: A Post-hoc Analysis

**DOI:** 10.1055/a-1926-2367

**Published:** 2022-09-30

**Authors:** Lauré M. Fijen, Remy S. Petersen, Joost C. M. Meijers, Laura Bordone, Marcel Levi, Danny M. Cohn

**Affiliations:** 1 Department of Vascular Medicine, Amsterdam Cardiovascular Sciences, Amsterdam UMC, University of Amsterdam, Amsterdam, The Netherlands; 2 Department of Experimental Vascular Medicine, Amsterdam Cardiovascular Sciences, Amsterdam UMC, University of Amsterdam, Amsterdam, The Netherlands; 3 Department of Molecular Hematology, Sanquin Research, Amsterdam, The Netherlands; 4 Ionis Pharmaceuticals, Carlsbad, California, United States

**Fig. 1:**
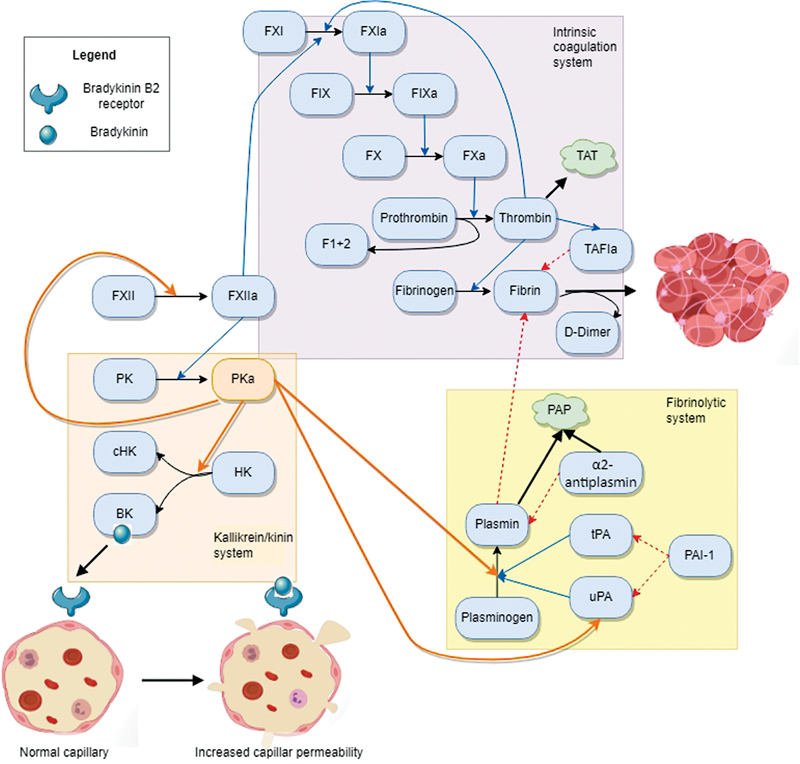
Overview of the functions of plasma kallikrein in the kallikrein/kinin, intrinsic coagulation, and fibrinolysis systems. Activated factor XII (FXIIa) converts plasma prekallikrein (PK) into plasma kallikrein (PKa). PKa cleaves high-molecular-weight kininogen (HK), resulting in cleaved HK (cHK) and bradykinin (BK). BK binds to its receptor on endothelial cells, leading to vascular leakage and thus angioedema. PKa also activates factor XII (FXII) which activates the intrinsic coagulation cascade, starting with factor XI (FXI), which becomes activated FXI (FXIa). FXIa converts factor X (FX) into activated FX (FXa), which converts prothrombin into thrombin and the residual product prothrombin fragment 1 + 2. Thrombin converts fibrinogen into fibrin, which forms blood clots together with blood cells and platelets. Thrombin is inhibited by the formation of complexes with antithrombin (TAT) and activates thrombin-activatable fibrinolysis inhibitor (TAFIa), which protects fibrin from being degraded by the fibrinolytic system. PKa also activates urokinase-type plasminogen activator (uPA), which, together with tissue-type plasminogen activator (tPA), is inhibited by plasminogen activator inhibitor-1 (PAI-1). Both plasminogen activators convert plasminogen into plasmin. Finally, PKa activates plasminogen as well. Plasmin degrades fibrin thereby releasing D-dimers. Plasmin is inhibited by α2-antiplasmin thereby generating plasmin–α2-antiplasmin complexes (PAP).

Hereditary angioedema (HAE) is characterized by recurrent mucosal and cutaneous swellings, resulting from excessive bradykinin generation, which is the end product of the kallikrein/kinin system. 1 2 HAE predominantly occurs in patients with congenital C1-inhibitor deficiency. C1-inhibitor controls the activation of factor XII (FXII) and plasma prekallikrein (PK). Notably, bradykinin-mediated HAE is also described in patients with normal levels and functionality of C1-inhibitor. 3 Inhibition of PK is currently being investigated as a prophylactic treatment in HAE. Activated FXII converts PK into plasma kallikrein (PKa), which cleaves high-molecular-weight kininogen (HK) thereby liberating bradykinin. PKa can activate FXII, plasminogen, and urokinase-type plasminogen activator. 4 During HAE attacks, both the coagulation cascade and fibrinolytic system seem to be activated as evidenced by elevated prothrombin fragment 1 + 2 and D-dimer levels. 5 Either way, HAE patients do not have a prothrombotic tendency. 6 Fig. 1 gives an overview of the functions of PKa in the kallikrein/kinin, intrinsic coagulation, and fibrinolysis systems. Congenital PK deficiency is a very rare condition that is presumed to be asymptomatic, but has been linked to increased risk of thrombotic events. 7 8 9 10 Congenital PK deficiency is usually detected when coagulation assays are performed, as the activated partial thromboplastin time is prolonged in the absence of PK. 6 11 The critical roles PK play in the kallikrein/kinin system and in in vitro coagulation are well known, but paradoxically PK does not contribute to in vivo hemostasis. 12 The link between reduced levels of PK and thrombotic risk is less well established. The validity of the claim that PK deficiency increases thrombotic risks stated in the previously mentioned case series is hampered by the lack of adequate control groups and the risks of both selection and publication biases. It is important to note that the majority of individuals with PK deficiency are presumed to go unrecognized given its largely asymptomatic nature. Thus, the occurrence of cardiovascular or thrombotic events in these subjects may have accounted for coagulation assays that revealed rare observations, which are then more likely to be published. Animal studies contradict the hypothesis of increased thrombotic risk in the absence of PK. 13 14 15 However, if an increased thrombotic risk of PK deficiency does exist, this can be caused by either enhanced clot formation via the intrinsic coagulation cascade or via decreased fibrinolytic activity. Increased thrombin generation could theoretically be caused by enhanced intrinsic factor XI (FXI) activation due to increased binding to HK, as the latter protein is less bound to PKa in this scenario. 16 Elevated FXI levels are associated with an increased risk of arterial and venous thromboses. 17 18 19 20 However, *KLKB1 ^−/−^* mice have similar FXI plasma levels compared with wild-type mice. 13

In this letter, we present the results of coagulation and fibrinolytic activity assays in samples obtained from a phase 2 trial in 22 HAE patients before and after 4 months (hereinafter referred to as follow-up) of treatment with either 80 mg PK antisense oligonucleotides (donidalorsen) or placebo. 1 At follow-up the median percent reduction from baseline in the donidalorsen group in PK levels was 75% (range: 36–94%) and the silica-based activated partial thromboplastin times remained within reference values. Coagulation and fibrinolytic activity markers at follow-up were compared with baseline and placebo-treated patients and the results are shown in Table 1 . All generic and specific assays measured at follow-up in the donidalorsen group were comparable with baseline. None of the concentrations or activities in the donidalorsen group differed significantly from the placebo group after Holm–Bonferroni correction. A sensitivity analysis of all outcome parameters in the randomized study population did not reveal any statistically significant changes compared with baseline or placebo either.

**Table 1:** Coagulation and fibrinolysis markers

Laboratory tests	Reference values	Donidalorsen baseline	Donidalorsen follow-up	Delta donidalorsen (follow-up minus baseline)	*p* -Value comparison, donidalorsen baseline and follow-up	Placebo baseline	Placebo follow-up	Delta placebo (follow-up minus baseline)	*p-* Value comparison, delta donidalorsen and delta placebo
CT lag time (min), *mean (SD)*	1.5–3.2	3.0 (0.6)	3.0 (0.6)	−0.0 (0.3)	1.00	3.4 (0.7)	3.1 (0.6)	−0.2 (0.5)	1.00
CT peak thrombin (%), *mean (SD)*	63–154	109 (34)	129 (32)	21 (27)	0.24	96 (22)	112 (27)	8 (15)	1.00
CT ETP (%), *mean (SD)*	61–146	98 (16)	108 (23)	9 (20)	1.00	104 (30)	107 (13)	1 (21)	1.00
Factor XI activity (%), *mean (SD)*	67–149	110 (31)	115 (27)	5 (20)	1.00	105 (28)	110 (27)	5 (21)	1.00
Prothrombin fragment 1 + 2 (pMol/L), *median (IQR)*	53–271	251 (186, 406)	161 (119, 259)	−71 (−183, −23)	0.64	225 (177, 267)	189 (163, 267)	−8 (−64, 5)	1.00
TAT complexes (µg/L), *median (IQR)*	<4.6	2.6 (2.2, 5.6)	1.9 (1.7, 2.3)	−0.2 (−4.1, 0.4)	1.00	2.3 (2.0, 2.9)	2.3 (2.0, 3.2)	0.0 (−0.1, 0.1)	1.00
HK activity (%), *median (IQR)*	60–130	87 (78, 97)	93 (84, 105)	7 (0, 15)	0.42	101 (84, 120)	92 (82, 110)	6 (−9, 16)	1.00
nCLT (%), *median (IQR)*	50–150	87 (79, 96)	93 (82, 108)	6 (1, 10)	1.00	87 (74, 106)	94 (70, 111)	−6 (−8, 0)	1.00
D-dimer (µg/mL), *median (IQR)*	<0.50	0.65 (0.44, 2.80)	0.36 (0.27, 0.55)	−0.20 (−2.25, −0.03)	0.40	0.36 (0.36, 0.68)	0.52 (0.43, 0.54)	0.10 (0.01, 0.16)	0.64
Plasminogen activity (%), *mean (SD)*	75–150	116 (25)	125 (21)	8 (16)	1.00	107 (23)	109 (25)	3 (19)	1.00
PAP complexes (ng/mL), *median (IQR)*	0–514	533 (276, 1,156)	328 (187, 381)	−75 (−741, 7)	0.79	237 (201, 328)	212 (191, 257)	−24 (−73, 16)	1.00
α2-antiplasmin activity (%), *mean (SD)*	80–120	122 (10)	127 (10)	7 (10)	0.41	116 (16)	121 (11)	5 (12)	1.00

Abbreviations: CT, calibrated automated thrombogram; HK, high-molecular-weight kininogen; IQR, interquartile range; nCLT, normalized clot lysis time; PAP, plasmin–α2-antiplasmin; SD, standard deviation; TAT, thrombin–antithrombin.

Note: Assessments at baseline and after 16 weeks of monthly treatment with 80 mg donidalorsen or placebo. *p* -Values are adjusted for multiple comparisons with the Holm–Bonferroni method. *n* is 16 for all donidalorsen assessments except for baseline nCLT and follow-up TAT where *n* is 14, and baseline α2-antiplasmin and D-dimer and all baseline CT parameters where *n* is 15. *n* is 6 for all placebo assessments except for all baseline CT parameters and baseline nCLT where *n* is 5.

We conclude that partial PK reduction of approximately 75% in HAE patients, albeit very effective in reducing attack frequency, does not translate into an increased coagulation activity or a decreased fibrinolytic activity. From these observations, one could infer that the thrombotic risk is not increased with PK deficiency. Our results are consistent with in vivo experiments in mice, as well as findings in HAE patients treated with lanadelumab. 13 14 15 21 The contrast in our findings with the suggested increased thrombotic risk postulated by Girolami et al and Barco et al 7 8 9 10 may be explained by methodological limitations or the more pronounced decrease of PK levels in patients with a congenital deficiency. We did not observe a decrease in HK activity after targeted PK reduction. This has not been previously investigated, but conversely a congenital HK deficiency has been reported to be associated with lower PK levels. 22 23 Binding to HK may protect PKa from inactivation or clearance. 24 25 Alternatively, congenital HK deficiency may be genetically linked to inherited PK deficiency. Our study has several notable strengths. We made paired comparisons between baseline and follow-up measurements in the same individuals. Additionally, we compared our results to an adequate control group of patients with the same condition treated with placebo. Another benefit is that we assessed both global measurements of the thrombin-forming and fibrinolytic systems, as well as a more detailed examination of several crucial enzymes and complexes within these systems. A limitation due to the restricted use of remaining plasma samples from a previously completed trial was the insufficient material available for all analyses in all patients. The samples which lacked adequate data were missing at random and are thus not expected to have influenced the validity of our results. A potential limitation of this study was the approximately 75% reductions in PK levels. This has previously been shown to be sufficient for decreasing angioedema attack rates 1 and we contributed with our study to the growing number of evidence that this amount of PK reduction does not increase thrombotic risk in HAE patients. However, we acknowledge that in congenital PK deficiency the PK levels are lower than in our study, meaning that our results cannot be extrapolated one-to-one to the thrombotic risk in patients with congenital PK deficiency.

In summary, our results do not demonstrate a procoagulant state in patients with approximately 75% reduced PK levels. This questions the earlier reported link between PK deficiency and increased thrombotic risk. In addition, we showed that HK activity, FXI activity, and plasminogen activity are not hampered by significantly reduced PK levels.
